# On Food Adulterations

**Published:** 1859-07

**Authors:** John Scoffern

**Affiliations:** London. Late Professor of Chemistry at the Aldersgate School of Medicine.


### ARTICLE XIV.

*On Food Adulterations.* By JOHN SCOFFERN, M. D., London. Late Professor of Chemistry at the Aldersgate School of Medicine.

No evils are so pernicious as cumulative evils ; and paradoxical though the assertion may seem, the aggregate result of a cumulative evil is the more considerable as the evil itself is small. One large dose of arsenic or corrosive sublimate involves a contingency of sufficient danger ; but such accidents are fortunately rare, and the subject of arsenical or mercurial poisoning would at least not have to lay to the charge of either of these poisons the quality of insidious nocuity. To an individual of ordinary intelligence, the symptoms of poisoning would, in nearly every case of this kind, be evidence enough ; and few indeed would be the instances of false diagnosis to a medical man. Far otherwise is it when noxious agents are ingested day by day in articles of food or drink. Take, for example, an instance of insidious lead poisoning. It is a fact too popularly known for comment to be necessary, that water, under certain conditions, acquires the property of dissolving, and holding in solution, the metal, lead ; whereas, under other circumstances, water may be transmitted through leaden pipes, and stored in leaden vessels with perfect impunity. The conditions of this safety and this danger will be fully explained hereafter. I so far anticipate the subject now, to illustrate the case of cumulative and insidious detriment to health by reference to the continuous ingestion of small doses of lead. Let us assume the existence of a pump with leaden pipes descending into a well, that well containing water of the variety which dissolves, and holds in solution, lead. It may be that the quantity of metal dissolved is so trivial that the water shall be altogether insipid ; and that one draught, or two, or ten, would be as harmless as the same water might have been had lead never come into contact with it ;—yet let a draught of such plumbiferous water be swallowed day by day for a continuous period, and the direst results almost necessarily follow. Firstly, vague symptoms of lassitude and general depression seize the members of a family thus circumstanced ; colic then follows, then paralysis, and finally death, if discovery of the causes, and succour, be not at hand.

Perhaps there can be no better illustration of the danger to be apprehended from the cumulative effects of continuous small doses of a noxious body, than is furnished by the case to which the reader's attention has been directed. That case will be readily seen to present the type of many others, differing in the degree of course, though corresponding in the general quality of insidious nocuity. It is the type of the insidious evils attendant on the ingestion of adulterated food and medicine. Perhaps the adulteration of medicines is the graver matter of the two—seeing, that whilst food is eaten by people in both health and disease, medicines especially concern the latter state. The stomach of an individual in health is an organ of wonderful endurance—of great accommodation. The usages of civilized society are such, that what with elaborate dishes and condiments, hotdrinks and spirituous stimulants, the stomach is accustomed to such an amount of ill-treatment, that the ingestion of many substances, themselves noxious, are scarcely more noxious than the normal ill-treatment which a civilized stomach is educated to bear. Practically, therefore, the ingestion of such noxious bodies may scarcely eventuate in a perceptible result. Far otherwise is it in the case of medicines taken into the stomach of one whose susceptibilities are feverishly aroused, and his senses morbidly sharpened. Any unwonted shock to the system, any error of calculation in the dose of a medicine, or (what amounts to the same thing) in its calculated power—whether as to degree or to kind—may act the part of the direst poison, and kill at once, though an influence of precisely the same kind, and degree, might count for nothing to a person in health.

Although it be not easy to draw a line of strict demarcation between food and medicines, considering the easy gradations by which one class merges into the other ; nevertheless the object of the succeeding pages will be to deal, as exclusively as possible, with the subject of adulterated food.

### THE TERM ADULTERATION DEFINED.

All who have attentively read the minutes of evidence tendered before Mr. Scholefield's committee on adulterations, cannot fail to have been struck with the ill-defined ideas attached to the word, adulteration, by the major part of the individuals examined. Putting aside the grave issues involved, it is somewhat amusing to remark how extremely shocked many gentlemen were at the words adulteration, contamination, impurity, &c. ; even while not merely explaining, or palliating, the acts to which such words applied but openly defending them. It is to be presumed that, after the mass of evidence tendered, some legislative enactments will follow, having for their object the prevention of food adulteration ; but it is a matter of great regret that some clear definition of what is meant by adulteration, contamination, impurity, &c.—clear, that is to say, in a legislative sense— has not yet been arrived at.

Various as were the shades of opinion manifested on this important subject by the different witnesses examined, the aggregate of testimony leans towards the adoption of what I cannot but regard as a fundamental error, viz. the adoption of the idea of nocuity as necessary to constitute an adulteration, in the legislative sense, of an article of food or drink. Now, unquestionably the idea of nocuity is intimately connected with the idea of adulteration or contamination of vital ingesta ; but the logical precision of philosophy is violated, and the application of that philosophy will be rendered abortive, by adopting a definition of purity, or contamination, based upon a previous determination of nocuity, or innocuity. The discrepancy of medical opinion as to the relative innocence or nocuity of articles of food is proverbial ; and not more proverbial than easily explicable—the fact being that in by far the greater number of instances, no absolute rule admits of being laid down. If human constitutions and idiosyncrasies were precisely similar, some correct deductions might be arrived at ; but the physical temperament of our bodies is no less various than the traits of human features. Articles of food which agree perfectly with some constitutions, disagree even to the extent of being poisonous with others. I know an individual who invariably suffers from cutaneous eruption, of erysipelatous character, after partaking of crab ; and the instances are frequent of persons who dare not eat some particular kind of fish ; amongst which salmon is conspicuous Here are types and indications, then, of the interminable fallacies which would result from the adoption of the idea of innocuity or nocuity as a basis of definition for the terms, adulteration, contamination, impurity, &c. When to organic and radical fallacy, which attachcs to a definition connected with the preceding ideas, and based upon them, are added the bias of interest and the disturbing influences of commercial expediency—when these disturbing elementsare reflected on, the conclusion is inevitably arrived at, that a thing will be pronounced noxious or innocuous according to the interests involved.

A great error has been committed, not only by the public generally, but also by professional men and legislators, in understanding the word impurity and its congeners in an absolute, instead of a relative, sense—than which nothing can be more unphilosophical. Hence, the words impurity and adulteration, as applied to articles of food, have come to stand for something injurious to the human organism. If food were alone in the category of adulterated articles, there might not practically be much objection to the acceptance of this popular notion, though in any case it is based on a secondary and specific quality—a basis generally devoid of the most correct elements of scientific classification.

In attaching a limiting significance to the terms, adulteration, contamination, and their congeners, it would be as unphilosophical as injudicious to alter their meaning for every class of bodies. Whatever definition of the terms be held to suffice in the case of articles of food, those terms should be sufficiently general to include all other cases of adulteration and contamination. Accepting this postulate, we must of course banish the ideas of nocuity and innocuity to the human organism, and acknowledge, as our foundation of the idea of adulteration, the principle of deviation from a standard. In proportion as the idea is embraced, so does the subject of adulterations become more clear, more succinct, more easily cognizable to the legislature; in proportion as this idea is departed from, so does the subject become more vague and undefined. Adopting this idea on its abstract merits, and contemplating the subject of adulterations in accordance with it, we shall soon find that these abstract merits are associated with an important practical issue. The premises once accepted that every deviation from a given standard is to be recognized as an impurity, adulteration, or contamination, we are soon led to the sound dictum of practical morality, that a purchaser should have that which he demands—if not an interdicted thing; and that any deviation is a fraud, a contamination, or adulteration.

*Substances Present in Minute or Infinitesimal Quantity.*—Every person acquainted with chemistry is aware that many substances are discovered in places, and under circumstances, where they would be little suspected. The chemist is perfectly well aware that he cannot take a feather, or a piece of flannel, however old, and worn, and washed the latter may be, without discovering in both evident traces of sulphur. Equally well the chemist knows that gold, scarce though it be as to gross amount, is one of the most extensively diffused of all bodies; not to mention its wide mineral distribution, and its presence in some vegetables; according to Dr. Percy gold is found in all oceanic waters which have come under his examination. In like manner, the chemist is aware that it would be impossible for him to subject any specimen of bread to analysis without discovering in it traces of various metals. These indications furnish only particular cases of the general proposition, that without the idea of a limit the most incorrect notions will arise from a literal acceptation of chemical expressions. Let it be assumed that a person sends a specimen of bread to a chemist for examination; the chemist subjects it to the scrutiny of his art, and replies that amongst other constituents he finds in it lime, as he assuredly would also find sulphur, phosphorus, and other bodies, the names of which are equally startling. How incorrect would be the resulting notions created in the mind of one ignorant of chemistry! Much of this kind of alarm has actually arisen in the public mind, owing to misapprehension respecting certain bodies, the presence of which is only true to the limit.

As a natural consequence of our free institutions, and our independence of action, the deceptive tricks of contamination, have been perhaps carried to a greater extent in these isles than elsewhere; nevertheless it cannot be denied that the subject has been lately brought before the public too much in the spirit of partizanship for the interests of abstract truth to be well subserved. Unfortunately, in this matter as in many others of cognate nature, the growing evil of purchased scientific special pleading has too often taken the place of scientific evidence; and thus, much information that might have been temperately and profitably conveyed to the public for their own protection, has been wilfully distorted by high-wrought deceptive statements, into *nearly* the semblance and *quite* the significance, of positive untruth.

As an example of this sort of scientific deception, the subject of aluminized bread may be adduced. Whether the presence of alum in bread be injurious to the human organism, or the contrary, I shall not discuss here, this being a secondary issue, not intimately connected with the topic to which I purpose to advert. Injurious or not injurious, the addition of alum to bread involves the addition of that which is foreign to the standard of bread as ordinarily accepted, and which, *pro tanto*, constitutes an adulteration or contamination. Reviewing the scientific evidence which has taken place on this subject, it will have been noticed, that not only is the question of the nocuity or innocuity of alum still open to the greatest doubt, which, indeed, may well be as the consequence of honest difference of opinion, but the very presence or absence of alum in bread, to the original materials of which alum was added, has been debated by opposing scientific evidence in a way calculated to mislead the public. Those scientific witnesses who aver that alum, though added to the materials of bread, does not exist in the bread itself, can hardly be said to have acted up to the poor standard of morality chosen by themselves; they can hardly be said to have been true to their word. Even assuming, as they desire to assume, that alum—a salt made up of potash, alumina, and water, loses its water by the amount of heat employed in the operation of bread-baking, (a most improbable assumption, by the way,) still it is hardly true to the letter to assert that alum minus water is no longer alum. In spirit, so egregiously untrue is the expression, that it is perhaps even worse in its consequences than a falsehood pronounced as such, because it is more insidious.

With every desire that some efficient legislative provision should exist for securing the purity of things in general, but more especially of articles of medicine and alimentary ingesta, I cannot but think that the subject of aluminized bread has occupied a specific attention which might have been more profitably directed to the subject of adulterations generally. To my appreciation, the point has never yet been satisfactorily made out that alum, in the quantities ordinarily used by bakers (and without expressing this limitation, all discussion of the subject would be absurd,) is more injurious than common salt.

*Legislative Measures having reference to the Adulteration of Ingesta.*—The principle of *caveat emptor* is one peculiarly congenial to the British character and British institutions. The jealousy of our race, in respect of all that relates to interference with the freedom of the subject, is, perhaps, the most remarkable trait in our political aspect. Amidst much that is good, traceable to the unrestrained expression of this self-dependent principle, there is unquestionably something of evil; and of the latter, the subject of adulterated ingesta furnishes an apt example. By the term ingesta, we may for the present understand not only articles of food and drink, but of medicines also—anything, in point of fact, which is taken into the stomach.

Now will it be evident, on the slightest inspection, that the principle of *caveat emptor* has not, *cannot* have the remotest significance in many cases of the purchase of articles of food; and still more emphatically does the remark apply to the majority of cases involving the purchase of medicines. Limiting our present observations to the case of the purchase of food, it will be evident that the means or power of discrimination at the purchaser's command are, for the most part, of the slightest and most inefficient description. Debarred from the evidence of chemical and microscopic examination, the purchaser for the most part, has no other rule of guidance than that furnished by the direct exercise of his senses ; but this direct application of the senses is, in the majority of instances, and those of the greatest importance, totally inoperative. No person, by the direct application of the senses, could discover the presence of lead in wine, or cider, or sugar, though the lead might be present in a highly dangerous quantity ; no one, by the direct application of the senses, could prove the existence of copper in pickles or confectionery, though copper might be present to a dangerous extent. These examples, involving the presence of bodies confessedly noxious to the animal organism, have been purposely chosen for the sake of illustrating the non-applicability of the dictum *caveat emptor* in extreme cases. Were we to investigate the instances of adulteration not involving the presence of bodies confessedly injurious to health, examples might be found almost *ad infinitum.*

Supposing, then, the determination to be arrived at that some legislative supervision shall take place, with the object of protecting the public against falsifications, against the presence of which they cannot protect themselves, the important question arises, how is this supervision to be accomplished? Shall the supervision be exercised by the chemical department of the inland revenue, or by sanitary officers to be specially created? Shall there be a metropolitan board of examination, or shall each district have its own local board? At whose instigation shall the examination of the materials of human ingesta take place? And lastly, who shall assume the functions of prosecutor? These points have been much discussed, without any definite result having been as yet arrived at.

In these general remarks, although exception has been taken to that definition of the terms impurity, contamination, adulteration, &c., which presupposes the presence of something injurious to health, I would by no means wish the reader to understand that the hygienic point of view is not the most important from which the subject of public bromatology can be contemplated. Nevertheless, it seems unphilosophical, and therefore highly objectionable, that the previous conditions of nocuity and innocuity should be involved in a definition—conditions in respect of which there is often the widest divergency of opinion, when the far simpler, far more philosophical rule of guidance of giving the purchaser what he asks for, can be so easily adopted.

This is a rule of guidance which, in its principle and its application, is unlimited; whereas, if we adopt the principle of limiting the terms, adulteration, contamination, &c., by the previous assumption of the qualities of nocuity and innocuity, not only do we introduce a never-ending topic of dissention, but we narrow the scope to which legislative enactments bearing upon the subject can be applied.

Having premised these general remarks, I shall now proceed to set before the reader a short summary of the chief contaminations which are prone to exist in certain common articles of food, whether the result of accident or design.

